# First report of urinary tract infection caused by *Comamonas kerstersii* in a goat

**DOI:** 10.1186/s12917-021-02840-x

**Published:** 2021-03-25

**Authors:** Silvia Pavone, Roberto Rinoldo, Elisa Albini, Alessandro Fiorucci, Biagio Caponi, Anna Fratto, Elisabetta Manuali, Paola Papa, Chiara Francesca Magistrali

**Affiliations:** Istituto Zooprofilattico Sperimentale dell’Umbria e delle Marche ‘Togo Rosati’, Via G. Salvemini, 1, 06126 Perugia, Italy

**Keywords:** *Comamonas kerstersii*, Emerging pathogen, Goat, Pyelonephritis, Urinary tract infection

## Abstract

**Background:**

*Comamonas kerstersii* is rarely associated with infections in humans and has never been reported in animals until now.

**Case presentation:**

Herein, we describe a case of urinary tract infection caused by *C. kerstersii* in a young goat. A seven-month-old male goat showed lethargy, generalised weakness and anorexia and in the last hours before its death, severe depression, slight abdominal distention, ruminal stasis, and sternal recumbency. Grossly, multifocal haemorrhages in different organs and tissues, subcutaneous oedema and hydrocele, serous fluid with scattered fibrin deposition on the serosa of the abdominal organs and severe pyelonephritis with multifocal renal infarction were detected. Histopathological examination confirmed severe chronic active pyelonephritis with renal infarcts, multi-organ vasculitis and thrombosis suggestive of an infectious diseases of bacterial origin. The bacterium was identified using routine methods, matrix assisted laser desorption/ionisation time-of-flight mass spectrometry (MALDI-TOF-MS), and sequencing of the *gyrB* gene.

**Conclusions:**

To the best of our knowledge, this is the first report of *C. kerstersii* infection in animals (goat). Our findings support the possibility of *C. kerstersii* isolation from extraintestinal sites and suggest this organism as a possible cause of urinary tract infection.

## Background

*Comamonas kerstersii* is a gram-negative, non-fermenting, oxidase- and catalase-positive motile bacterium first described in 2003 [1]. The genus *Comamonas* originally contained *C. terrigena, C. testosteroni* (previously *Pseudomonas testosteroni*), and *C. acidovorans* (previously *P. acidovorans*) [2]. Now, *Comamonas* genus contains 17 species including *C. terrigena*, *C. aquatica*, *C. kerstersii*, *C. testosteroni*, *C. denitrificans*, *C. nitrativorans*, *C. koreensis*, and others [3]. Although they are ubiquitously distributed in the environment (soil and water), *Comamonas* sp. are rarely associated with infections in humans and have never been reported in animals until now. In particular, *C. kerstersii* has recently been associated with human invasive infections, such as abdominal and urinary tract infections, and bacteraemia [4–8].

Herein, we describe a case of urinary tract infection caused by *Comamonas kerstersii* in a young goat. To the best of our knowledge, this is the first report of *C. kerstersii* in animals.

## Case presentation

A post mortem examination was performed on a seven-month-old male goat with a history of lethargy, generalised weakness and anorexia and in the hours preceding its death, severe depression, slight abdominal distention, ruminal stasis, and sternal recumbence. The animal came from a farm in central Italy consisting of twenty adult goats (two males and eighteen females).

Histologic samples of organs with pathologic changes were collected and the tissues were fixed in 10 % neutral buffered formalin, embedded in paraffin wax, sectioned at 5 μm and stained with haematoxylin and eosin.

Spleen samples were collected for molecular analysis of the Border disease virus (BDV). Molecular genetic investigation was based on the 5′ untranslated region (UTR) coding. Intestinal, pulmonary, hepatic, and renal specimens were aseptically collected for bacteriological examination. Moreover, peritoneal swabs and urine samples obtained by cystocentesis were tested. The samples were spread plated on MacConkey agar, mannitol salt agar and blood agar plates for aerobic incubation at 37 °C for 3 days. Suspected colonies were identified using a MALDI-TOF MS instrument (Bruker Daltonics, Bremen, Germany) with Microflex LT Smart Biotyper with FlexControl Biotyper 3.4 software (Bruker Daltonics, Bremen, Germany). Identification was confirmed by sequencing the *gyrB* encoding for the B subunit of the DNA gyrase, using the primers described by Tayeb et al. [9]. Antibiotic susceptibility testing was performed using a commercially available MIC plate (COMPAN2F, Thermo Fisher Scientific, Waltham, USA). The performance of MIC testing was verified using *E. coli* ATCC 25,922 as a control strain, as recommended by Clinical & Laboratory Standards Institute (CLSI) [10]. The minimal inhibitory concentration (MIC) of ampicillin was further evaluated using a Liofilchem MIC strip (Liofilchem, Teramo, Italy). Briefly, a 1 McFarland standard-matched suspension from a 24-h pure culture on Brucella blood agar supplemented with 5 % horse red blood cells (Brucella agar, hemin, Sigma-Aldrich, Milan, Italy) was distributed on a Brucella blood agar plate with the MIC strip. The culture plate was incubated at 37 °C for 24-h and MIC determination was performed according to the manufacturer’s instructions. The CLSI breakpoints for ‘other non-Enterobacterales’ [10], when available, were used to interpret the MIC results as susceptible, intermediate and resistant. The MIC values for amoxicillin + clavulanic acid, ampicillin, cefazolin, penicillin, and ticarcillin were interpreted using pharmacokinetic/pharmacodynamic (PK/PD) values, according to the European Committee on Antimicrobial Susceptibility Testing (EUCAST) recommendations [11]. We investigated the presence of *strA*, *strB*, *tet(A), sul1, sul2*, and *bla*_OXA−1/4/30_, which have already been described in *C. kerstersii* [12]. Furthermore, we assessed the presence of other genes coding for resistance to beta-lactams and aminoglycosides using polymerase chain reaction (PCR) tests targeting *aac(6’)-Ib-cr*, *aadA*, *bla*_TEM−1/2_, *bla*_SHV−1_, *bla*_CTX−M−1_, *bla*_CTX−M−2_, *bla*_CTX−M−9_, *bla*_CTX−M−8_ and *bla*_CTX−M−25_, as previously described [13–20].

For anaerobic incubation, the samples were spread also on a blood agar plate placed in an anaerobic jar with AnaeroGen reagent (Thermo Fisher Scientific, Waltham, USA) and incubated at 37 °C for 18–24 h. Incubation was prolonged for two more days if no colonies appeared at 24 h.

Coprological examination was carried out using the FLOTAC technique on five grams of feces.

**Fig. 1:**
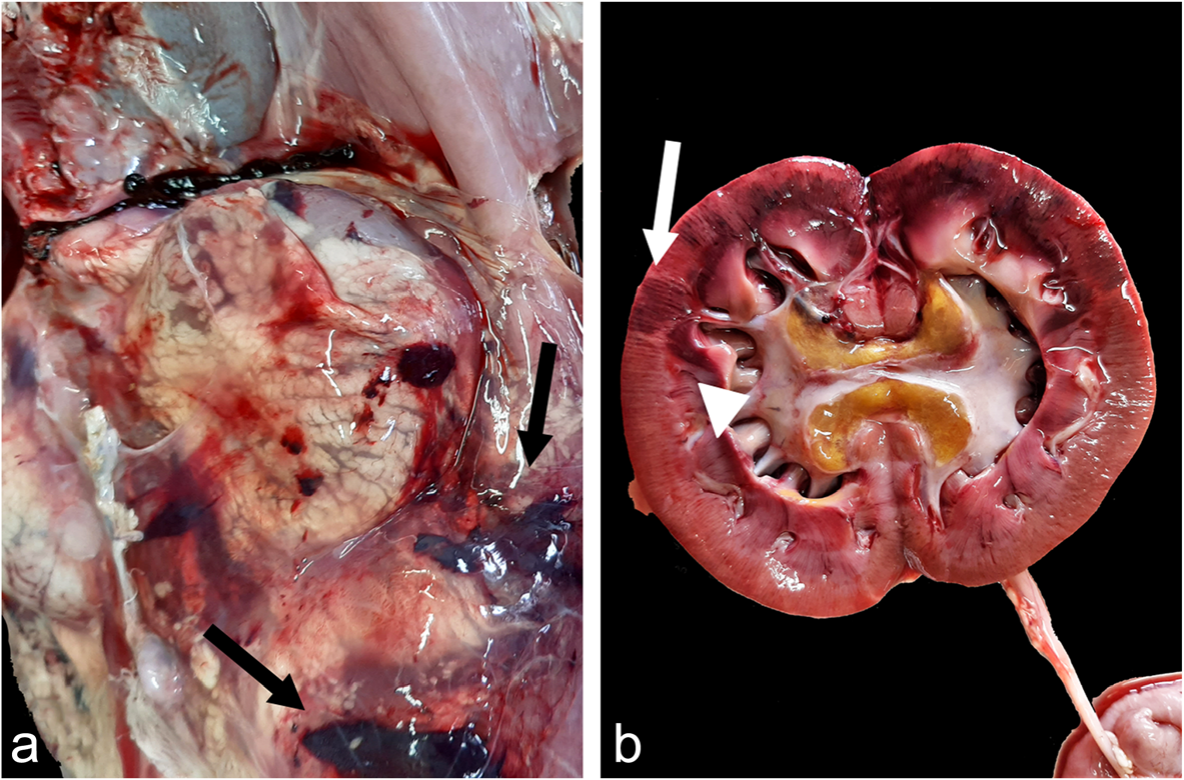
Photographs of gross lesions in the urinary tract of goat. **a** Oedema and hemorrhages (arrows) of perirenal adipose tissue in the left renal lodge. **b** Left kidney. Cortical discoloration with hemorrhagic wedge-shaped area (arrow) and pelvis characterized by greyish and yellowish tissue

At necropsy, the goat appeared in good condition (body condition score = 3) and in a good conservation status, but was dehydrated. Conjunctival, subcutaneous, and muscular haemorrhages associated with subcutaneous oedema and hydrocele were found. Within the abdominal cavity, there was moderate serous fluid with scattered fibrin deposition on the serosa of the abomasum, liver, and intestinal loops. Petechiae and ecchymosis were detected in the perirenal adipose tissue (Fig. 1a). The kidneys were swollen and markedly congested. On the cut surface, the renal cortex of the left kidney showed discolouration with pink-gray areas alternating with hemorrhagic wedge-shaped areas (renal infarction) (Fig. 1b). The pelvis showed yellowish and grey areas, and was oedematous and grossly free of obstruction. The contralateral kidney showed marked congestion and haemorrhages. No obstruction or apparent lesions on the mucosa of the ureters, bladder, or urethra were detected. Slightly cloudy and dark urine samples were observed. Congestion of the liver and catarrhal enteritis was also detected. In the thoracic cavity, the lungs were patchily congested and haemorrhagic; endocardial and epicardial haemorrhages were observed. No other macroscopic lesions were observed.

**Fig. 2:**
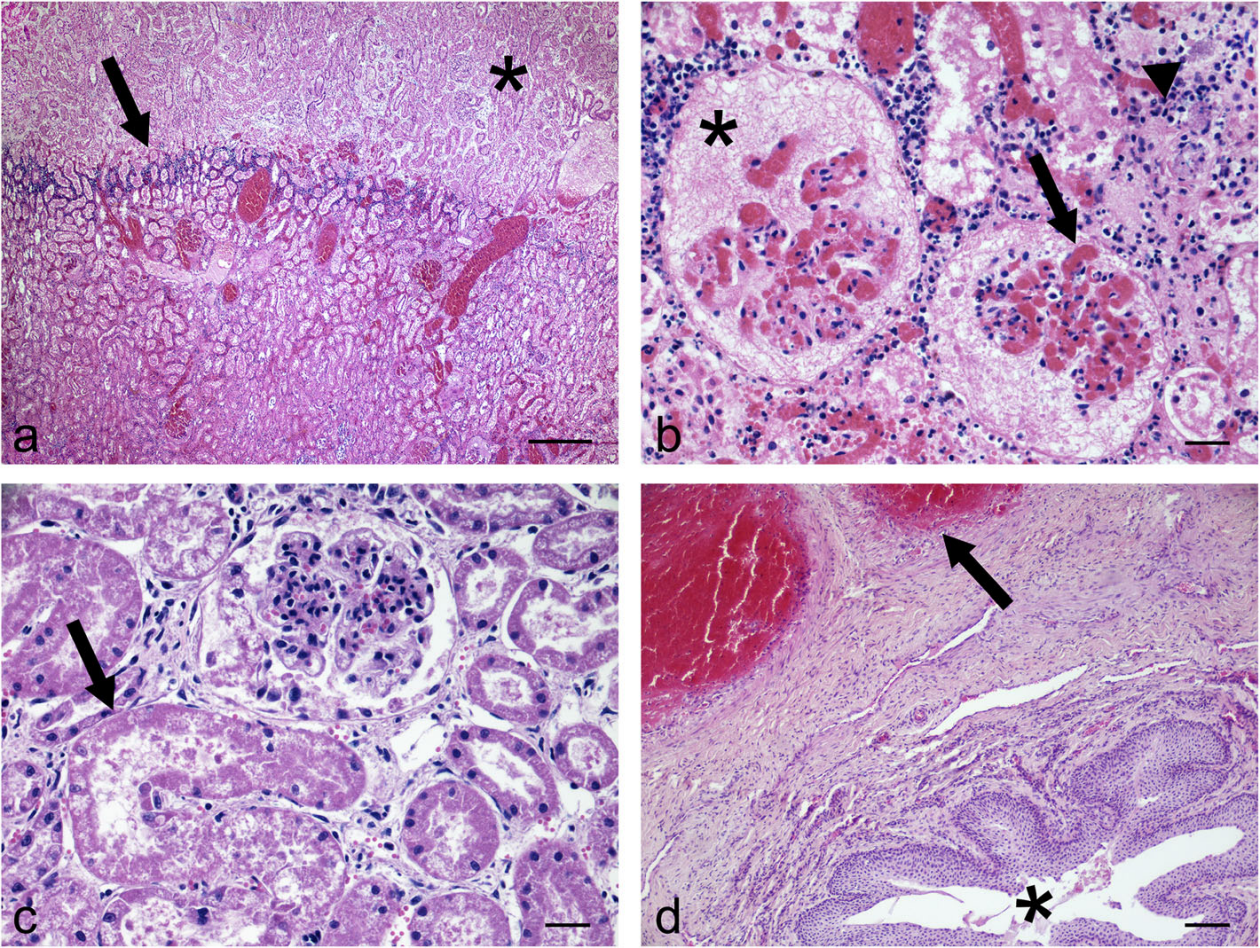
Histological changes in the urinary tract of goat. **a** Left kidney characterized by extensive cortical coagulation necrosis (asterisk) bounded by neutrophilic tubule-interstitial infiltration (arrow). Marked and diffuse vascular congestion. (Hematoxylin and eosin, Bar = 200). **b** Left kidney showed marked congestion and dilation of glomerular capillary loop (arrow) with glomerular collapse and accumulation of fibrin in Bowman’s space (asterisk). Interstitial neutrophilic inflammatory infiltrate and scattered foci of mineralization (arrowhead). (Hematoxylin and eosin, Bar = 25). **c** Right kidney showed glomerular collapse with accumulation of cellular debris in Bowman’s space and necrosis of renal tubules (arrow). (Hematoxylin and eosin, Bar = 25). **d** Vasculitis (arrow) of medium arteries of penile urethra (asterisk). (Hematoxylin and eosin, Bar = 100)

Histological examination of the left kidney showed large areas of cortical necrosis of glomeruli and tubules surrounded by tubule-interstitial infiltration with neutrophils (Fig. 2a and b). Marked congestion and dilation of the glomerular capillary loop with glomerular collapse and accumulation of fibrin and cellular debris in the Bowman’s space were observed (Fig. 2b). Extensive necrotic processes associated with abundant interstitial and intratubular neutrophilic infiltrates were detected in the renal pelvis; multifocal areas of interstitial fibrosis with scattered lymphocytes and plasma cells (chronic inflammation) can be found in the renal papillae. Vasculitis, fibrinoid necrosis, and thrombosis of the vessels of the renal capsule associated with severe oedema were detected. In the right renal tissue, there was glomerular collapse with an accumulation of cellular debris in the Bowman’s space, scattered necrotic tubules (Fig. 2c), and fibrosing, necrotizing, and lymphoplasmacellular and suppurative pyelitis (chronic-active inflammation). Severe vasculitis and fibrinoid necrosis of small to medium arteries of the penile urethra (Fig. 2d) associated with degenerative changes, and attenuation of the transitional epithelium of ureters, and marked interstitial oedema of peripheral connective and adipose tissue were observed. On the other hand, histological examination of the intestine showed moderate, subacute, diffuse lymphocytic and eosinophilic enteritis, whereas the lungs showed alveolar oedema, severe congestion of pulmonary capillaries and multifocal hemorrhages. Overall, the lesions were consistent with severe chronic active pyelonephritis with renal infarcts, multi-organ vasculitis, and thrombosis suggestive of infectious diseases of bacterial origin.

The bacteriological examinations showed negative results in pulmonary, intestinal and hepatic specimens, and from peritoneal swabs. From the renal specimen and the urine, we observed the growth of a smooth, light-grey, non-haemolytic colony in pure culture. The colony was identified as *Comamonas kerstersii* by MALDI-TOF MS with a high-confidence identification spectral score of (2.22) and high consistency interpretation (A), since the same species was identified as the second- and third-best match. Other species within the genus, *C. testosteroni* and *C. aquatica*, resulted in low identification score, 1.42 and 1.32, respectively. An alignment search for the *gyrB* sequence using the Basic Local Alignment Search Tool (BLAST) on the NCBI website revealed 99.07 % identity with the *gyrB* sequence of the *C. kerstersii* strain 8943 sequence (accession number CP020121.1), confirming the previous MALDI-TOF MS identification. The sequence of the *gyrB* gene of the *C. kestersii* described in this manuscript has been submitted to NCBI GenBank (GenBank accession no. MW349112).

The MIC values of the control strain were in accordance with the reference values reported by the CLSI [10]. For the *C. kerstesii* isolate, the following MICs (mg/L) were determined: amikacin, 8; amoxicillin + clavulanic acid, 0.25/0.12; ampicillin, ≥ 1; cephalothin, ≤ 2; cefazolin, ≤ 1; cefovecin, ≤ 0.25; cefoxitin, ≤ 2; cefpodixime, ≤ 2; ceftiofur, ≤ 0.25; chloramphenicol, ≤ 4; doxycycline ≤ 2; enrofloxacin, ≤ 0.25; gentamicin, 2; imipenem, ≤ 1; marbofloxacin, ≤ 0.25; penicillin, 2; ticarcillin, ≤ 8; ticarcillin + clavulanic acid, ≤ 8/2; trimethoprim + sulphamethoxazole, ≤ 0.5/9.5. According to the CLSI breakpoints [10], the isolate was classified as susceptible to amikacin, chloramphenicol, doxycycline, gentamicin, imipenem, ticarcillin + clavulanic acid and trimethoprim + sulphamethoxazole. Based on the PK/PD values [11], the isolate was not susceptible to penicillin and ampicillin, but susceptible to amoxicillin + clavulanic acid, cefazolin, and ticarcillin. No breakpoints were available for the other tested molecules from the same source. The strain tested positive for *strA* and *strB* genes that confer resistance to streptomycin, whereas other antibiotic-resistance genes were not found.

Copromicroscopic examination revealed 2064 coccidian oocysts per gram of faeces (o.p.g). On the other hand, negative results were obtained from molecular analysis of the BDV.

## Discussion and conclusions

To date, infections caused by *Comamonas kerstersii* have been reported only in humans. This infection is often associated with peritonitis resulting from the presence of a perforated appendix, diverticulosis, pelvic peritonitis, and bacteraemia [4–7]. More recently, an unusual case of urinary tract infection in a 5-year-old girl was reported [8].

Herein, we reported a case of chronic-active necrotising and suppurative pyelonephritis caused by *Comamonas kerstersii* isolated from both renal specimens and from urine, and identified with the use of MALDI-TOF MS. It is well known that the use of MALDI-TOF MS for routine bacterial identification has revolutionised microbiology, allowing the identification of infectious agents efficiently and quickly [8]. The growing use of this method in veterinary medicine is likely to allow to identify an increasing number of pathogens considered rare or never detected in animals.

The increase in *Comamonas kerstersii* isolation frequency from human infections in the literature and now the isolation in urinary tract infection in goat identifies it as an emerging pathogen. Since translocation from the digestive tract seems to be a predominant cause of infections by *C. kerstersii* [21], we looked for this microorganism in the intestinal content, but obtained negative results. Therefore, and in view of the highlighted renal histological pattern (medullary changes seemed to become chronic while the cortical lesions were yet in an acute phase), the ascending path represents the most likely route of infection in this case.

As previously suggested, *Comamonas kerstersii* could be an opportunistic pathogen or commensal in the digestive tract and appendix in humans [22]. In this case, the presence of *C. kerstersii* as the only microorganism isolated from the urinary tract of goat, with no apparent known cause of immunodeficiency disorders points to it as a primary pathogen; however, a severe coccidiosis found in the present goat may represent a cause of debilitation and potential predisposing factor for opportunistic bacterial infections.

According to CLSI breakpoints for ‘other non-Enterobacterales’ [10], our strain showed susceptibility to antimicrobials, but this finding should be interpreted with caution since no specific breakpoints for the *Comamonas* genus are available. We observed high MIC values for two aminoglycosides, gentamicin and amikacin, and the presence of two genes encoding resistance to streptomycin, another member of the same antibiotic class. The presence of these genes was detected in the same species by Jiang et al. [12]. Our isolate was also classified as non-susceptible to ampicillin and penicillin according to the EUCAST PK/PD breakpoints. There is a paucity of data on the susceptibility of *C. kerstersii* to antibiotics in the literature, although other authors have reported the susceptibility of *C. kerstersii* isolates to ampicillin [4, 7], suggesting that *C. kerstersii* is not naturally resistant to ampicillin. Further research is needed to investigate the genetic background of resistance to β-lactamase in this species. Indeed, the presence of class A β-lactamase has already been described in another member of the genus, *C. testosteronii* [23].

In conclusion, this report describes the first case of *Comamonas kerstersii* infection in animals (goat). Our findings support the possibility of *C. kerstersii* isolation from extraintestinal sites and suggest this organism as a possible cause of urinary tract infection.

## Data Availability

The datasets used and analyzed during the current study are available from the corresponding author on reasonable request.
